# Implant loosening following THA with S-ROM prosthesis and subtrochanteric osteotomy: Three case reports

**DOI:** 10.3389/fsurg.2022.1090067

**Published:** 2023-01-30

**Authors:** Yingkai Ma, Xinnan Ma, Shi Cheng, Songcen Lv, Xin Qi

**Affiliations:** ^1^ Department of Orthopedics, Second Affiliated Hospital of Harbin Medical University, Haerbin, China; ^2^ Department of Orthopaedic Surgery, The First Hospital of Jilin University, Changchun, China

**Keywords:** osteotomy under the lesser, prosthesis loosening, s-ROM, DDH, revision

## Abstract

Prosthesis loosening after THA is a rather common complication. For DDH patients with Crowe IV, the surgical risk and complexity is significant. THA with S-ROM prosthesis combined with subtrochanteric osteotomy is a common treatment. However, loosening of a modular femoral prosthesis (S-rom) is uncommon in THA and has a very low incidence. With modular prostheses distal prosthesis looseness are rarely reported. Non-union osteotomy is a common complication of subtrochanteric osteotomy. We report three patients with Crowe IV DDH who developed prosthesis loosening following THA with an S-ROM prosthesis and subtrochanteric osteotomy. We addressed the management of these patients and prosthesis loosening as likely underlying causes.

## Introduction

Developmental dysplasia of the hips (DDH) is a major contributor to secondary arthritis. Ultimately resulting in total hip arthroplasty (THA) to relieve pain and improve function (1, 2). DDH is classified according to the severity of the dislocation, which is most generally based on the Crowe rubric. THA for DDH differs from general surgery, as dictated by the anatomical abnormalities of the DDH. According to the situation of each patient, Clinicians must develop specific surgical plans. Since infancy, these patients exhibit pathological alterations of hip dislocation that are typically asymptomatic or not obvious. As the patient ages, the degenerative alterations become much worse and the symptoms become progressively more apparent. The main manifestations are as follows: The acetabulum becomes shallow, the opening is widened, the anterior medial wall of the acetabulum is weak, the posterior wall has enough bone mass, and the femoral head is gradually displaced upward to form a false acetabulum. The anteversion angle of the femoral neck grew, the greater trochanter of the femur shrank and shifted posteriorly, the femoral bone marrow cavity was narrowed, and the proximal one-third of the femur was bent forward. The joint capsule is hypertrophied and extended, with the lower portion adhering to the pelvic wall and preventing the femoral head from entering the true acetabulum. The abductor muscle is transverse, its function is weak, and the muscles around the joint are contracted. These pathological changes were more significant in patients with Crowe III and IV DDH.

The combination of total hip arthroplasty and femoral shortening osteotomy is a successful treatment for Crowe IV DDH. In contrast to traditional prostheses, S-ROM prostheses are typically used for patients with DDH, and postoperative recovery is satisfactory (3–6). Routine postoperative prosthesis loosening is a frequent complication of THA, and aseptic loosening is considered a leading cause of revision total hip arthroplasty (RTHR) (7). According to previous studies, osteolysis are the leading causes of hip revision (8). However, loosening after an S-ROM replacement is relatively rare.

Owing to the rarity of this particular loosening of a prosthesis, there is currently no clear management guideline, which must be managed based on the position and cause of the loosening. Extremely challenging revision surgery must address the stability of the prosthesis and hip joint following replacement. Even so, they present unique surgical concepts and medical difficulties. However, the loosening of prostheses following S-ROM replacement has not been investigated individually. This study describes three cases of aseptic distal prosthesis loosening following THA with an S-ROM prosthesis and subtrochanteric osteotomy. In addition, by reviewing published cases of loosening after replacement, this study elucidated the causes of loosening and surgical treatment options to achieve the best treatment results.

## Case series

The case study required informed consent for subjects to be included in the study.

This case study included three patients who underwent THA with an S-ROM prosthesis and subtrochanteric osteotomy for Crowe type IV DDH in accordance with the Declaration of Helsinki standards. All three patients experienced postoperative prosthesis loosening at different times. The primary symptoms following THA were local discomfort in the lateral thigh on the operating side, positive percussion pain, and aberrant thigh movement.

**Figure 1:**
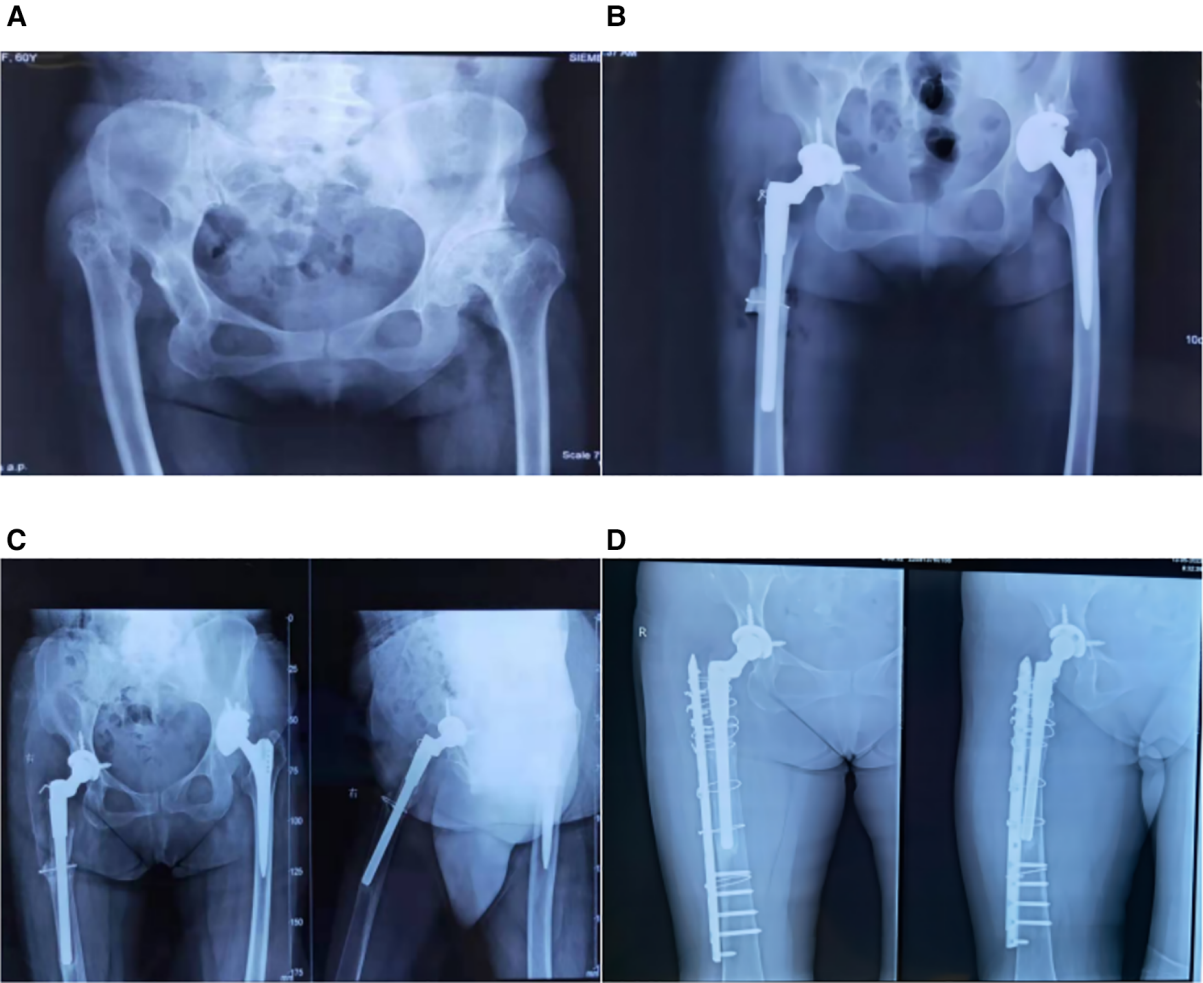
(**A**): radiographs of patient 1 prior to THA. On the right is Crowe IV, while on the left is Crowe II. (**B**): It displays patient 1′s hip radiograph following bilateral THA. (**C**): x-ray reexamined two years after the right THA shows loosening of the prosthesis. (**D**): It displays radiographs of the right hip of the patient after revision surgery.

Case 1: A 62-year-old female patient was observed for the first time in March 2017. She walked in a limp walking posture and complained of bilateral hip pain that had gradually worsened since 2008. A diagnosis of DDH was made. Figure 1A shows the preoperative imaging results of the patient. The patients were admitted to the hospital for complete preoperative examination and preparation for surgical treatment. Left total hip replacement (March 27, Smith & Nephew); right subtrochanteric osteotomy followed by total hip replacement (April 14, Johnson & Johnson S-ROM prosthesis) (Figure 1B). In October 2017, 5.5 months following surgery, soreness of the right thigh was noticed. The patient underwent an outpatient appointment for radiography and blood cell analysis. The CRP and blood sedimentation rate were normal. The patient was prescribed celecoxib capsules till July 2019 because of the patient's frequent and progressively severe pain. Anteroposterior and lateral femur radiographs were collected for evaluation (Figure 1C). The physical examination indicated lateral right thigh soreness, positive percussion pain, and aberrant activity in the middle and upper thirds of the right thigh. A diagnosis of loosening after total hip arthroplasty was made, and the patient was admitted for revision surgery. During the surgery, the proximal femur bone grew properly and did not loosen. According to the treatment of femoral prosthesis fracture or nonunion, a femoral window was opened at the distal loosening of the femoral stalk, and bone grafting was conducted at the femoral window. After osteotomy nonunion callus excision, bone grafting was conducted at the nonunion site; the femur was repaired with the titanium plate and screw, allogeneic bone plate reinforcing, and steel wire binding fixation (Figure 1D).

**Figure 2:**
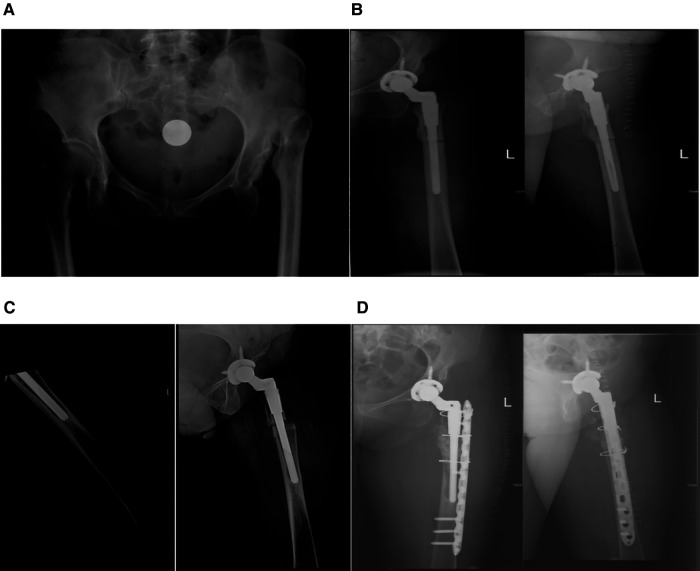
(**A**): anteroposterior pelvic x-ray with a diagnosis of left crowe IV DDH (left). (**B**): x-ray of initial subtrochanteric osteotomy with S-ROM prosthesis replacement. (**C**): After the patient fell, the distal end of the S-ROM prosthesis became loose, (**D**): x-ray of the left femur after revision of the left hip joint.

Case 2: A 59-year-old man with a 5-year history of hypertension and a 10-year history of myocardial infarction did not take regular medication to control his condition. The patient was diagnosed with Crowe IV DDH (Figure 2A). He received THA on his left side five years ago and recovered nicely. The imaging exam was as depicted in Figure 2B. The left femur was injured six months ago as a result of a fall while walking; nevertheless, no special treatment was administered, and the discomfort in the affected leg steadily intensified. An x-ray was performed (Figure 2C), and the patient was admitted for surgical treatment after being diagnosed with left hip prosthesis loosening (distal femur). Intraoperatively, with the greater trochanter of the femur as the centerpiece, a lateral hip joint incision is made, the skin and subcutaneous tissue are removed step by step, and then a broad tensor fascia and hip muscles are exposed to reveal the hip prosthesis. The prosthesis was dislocated. When we evaluated the healing of the femoral portion of the hip joint, we discovered that the proximal section of the S-ROM prosthesis fused entirely with the bone. The distal femur was examined, and it was discovered that the bone had not healed at the original osteotomy site; therefore, we transplanted bone around the distal chamber of the prosthesis and at the non-healed site. Titanium plates and screws were used to fix the femur, and wire was tied around the femur (Figure 2D).

**Figure 3:**
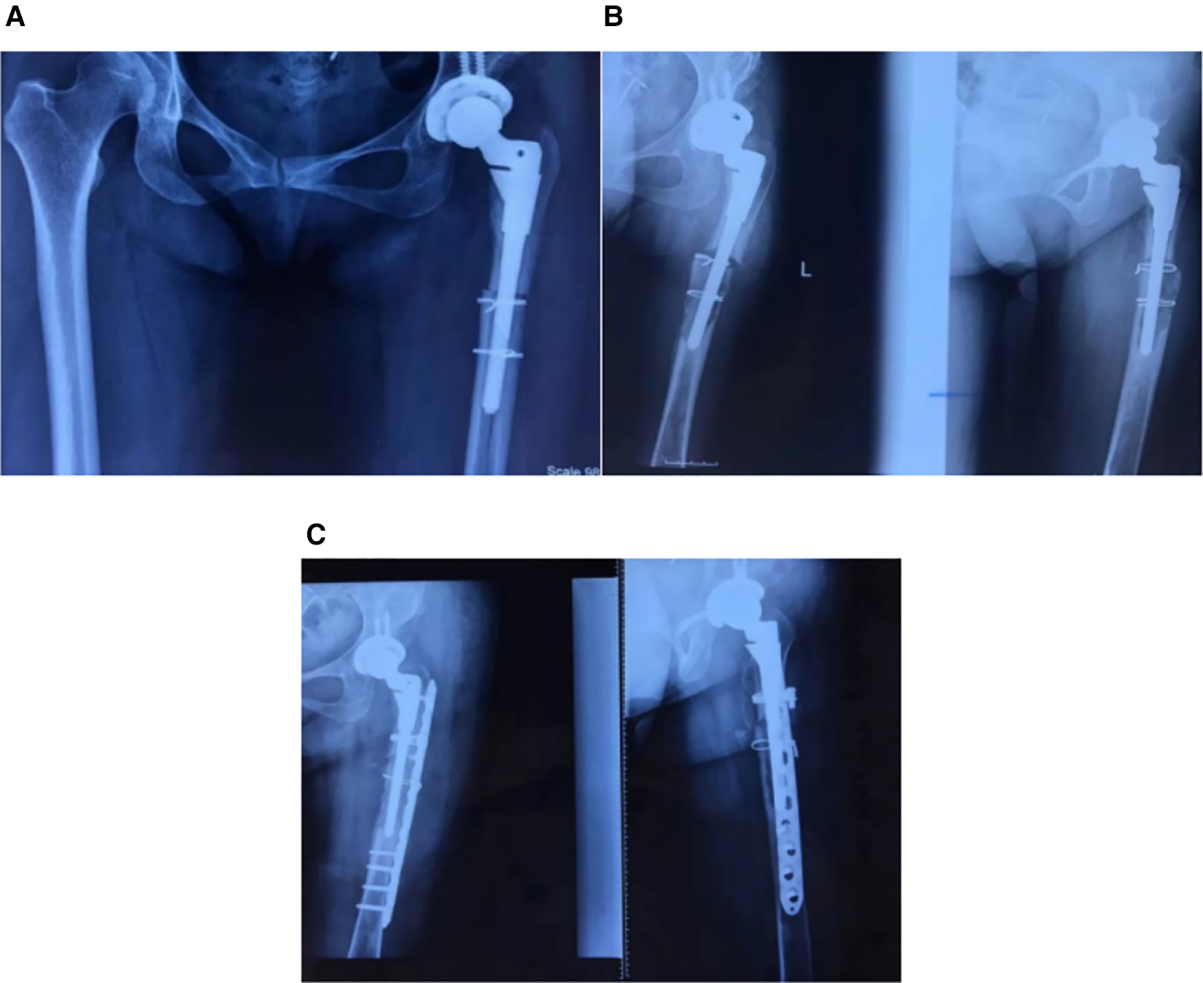
(**A**): anteroposterior radiographs of the pelvis after the patient's initial S-ROM prosthesis replacement are shown on the left, (**B**): radiographs of the hip joint with loose left hip prosthesis are shown on the right one year later. (**C**): Anterior and lateral femur radiation after revision of the femur.

Case 3: A 48-year-old woman diagnosed with left Crowe IV DDH underwent THA using an S-ROM prosthesis. Figure 3A depicts the imaging of the left hip joint after surgery. One year after the operation, pain and limited movement in the left limb arose, prompting a hospital imaging scan. Figure 3B depicts the x-ray of the patient's left femur one year after THA, and physical examination revealed soreness of the left lateral thigh and positive percussion pain. The patient was admitted for preoperative evaluation and surgical treatment preparation. The incision was made along the original incision, and intraoperative investigation revealed that the proximal femoral bone was well-ingrown and not loose. The distal end was next investigated, and the initial osteotomy site was found to be unhealed. Bone grafting was conducted around the prosthesis in the distal cavity, as well as at the unhealed location. The proximal portion of the femur was secured with single cortical screw, whereas the distal portion was treated using titanium plates and screws. The wire was tied around the femur (Figure 3C).

**Figure 4:**
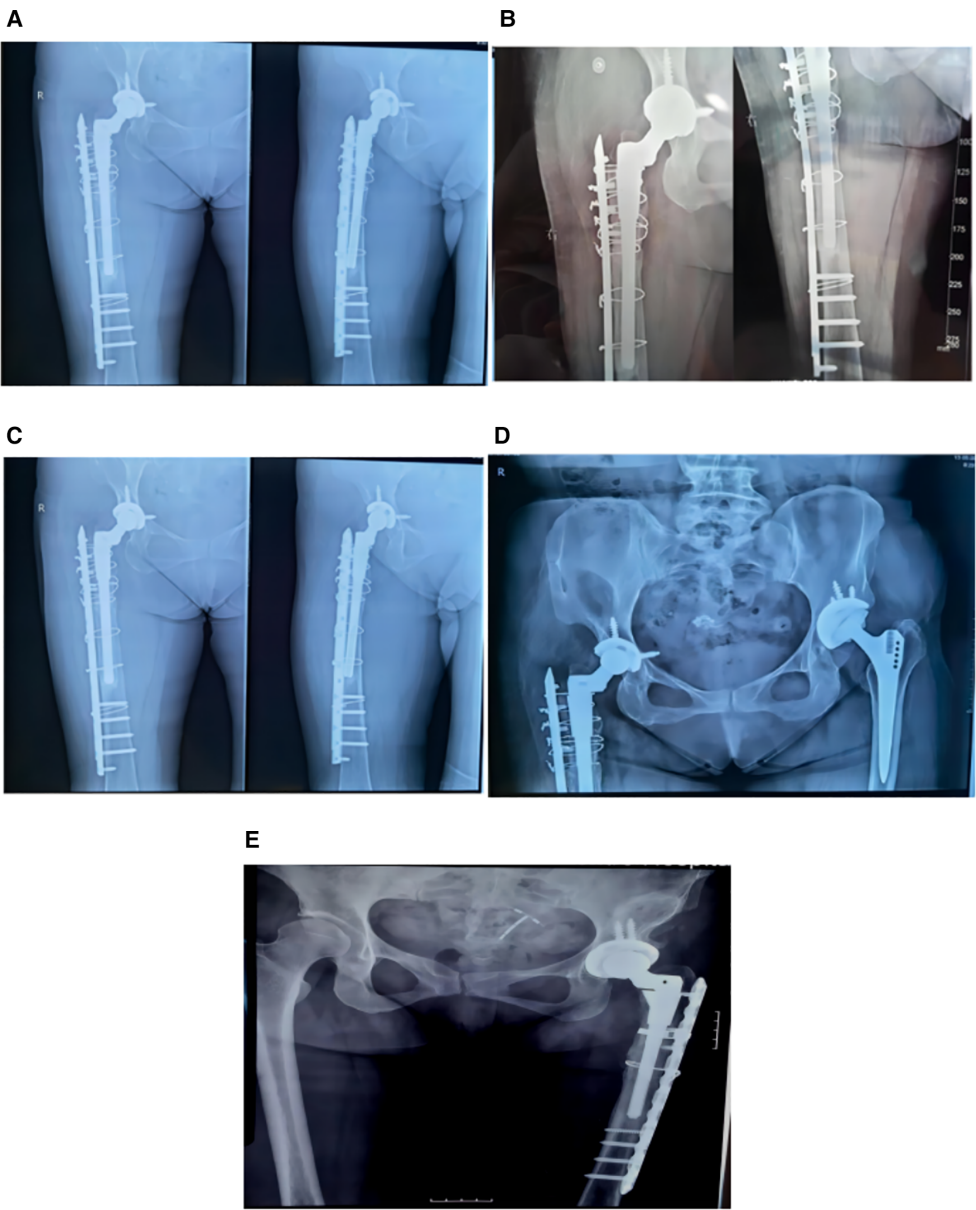
(**A**): the review results of patient 1 1 month after surgery; (**B**): the review results of patient 1 3 months after surgery. (**C**): anteroposterior femur of patient 1, 1 year after surgery; (**D**): anteroposterior pelvis of patient 1, 1 year after surgery. (**E**): The review results of patient 3 1 year after surgery.

All patients were subjected to postoperative forbidden activities, intravenous antibiotics, and preventative antithrombotic treatment(bid). Oral nsaids were administered, The drainage tube was withdrawn three days following surgery, Radiographic scans obtained postoperatively revealed that the hip joint's stability had been restored. After revision, the review was conducted at 3, 6, and 12 months postoperatively. that the hip implant loosening healed effectively (Figure 4). After six months of follow-up, all patients reported nearly no discomfort, were able to walk up and down stairs unassisted, and their Harris score after revision exceeded 85.

## Discussion

THA for Crowe type IV DDH is challenging (9). THA has had tremendous success in anatomy and postoperative function (10). However, the potential risks of THA include dislocation (11), leg length discrepancy (LLD), perioperative femoral fracture, nonunion at the osteotomy site, and nerve injury (12). Patients have considerable muscular injury, whereas patients with Crowe type IV DDH suffer thigh muscle atrophy due to long-term deformity (12), this increases the likelihood of postoperative complications. However, in patients with Crowe IV DDH with significant clinical symptoms and signs or in patients having revision surgery. The S-ROM prosthesis is frequently the best option because the limited bone marrow cavity of the femur can limit the penetration of non-modular stems (1). Lower limb length, femur forward tilt, and femur offset can be adjusted using a modular hip prosthesis. In addition, femoral shortening is necessary to prevent sciatic nerve injury in cases of more severe dysplasia (Crowe III or IV) with central dislocation of the hip joint or significant subluxation. Osteotomy aims to balance the leg length on the femoral side, reduce the height of the femoral head without causing damage to the sciatic nerve, and return the hip joint to its original center in a safe manner (13). THA combined with subtrochanteric osteotomy demonstrated positive effects in the DDH group (5). Osteotomy under the lesser trochanter can be performed using various techniques (14, 15), There have various types of osteotomies have been reported, such as V-shaped osteotomy (16), Z-osteotomy (17), step-cut osteotomy (18), chevron osteotomy (19) and transverse osteotomy. Chen et al. and Zeng et al. suggested that transverse osteotomy is better (9, 20). We performed subtrochanteric osteotomy 2 cm below the trochanteric. As the chosen type, transverse osteotomy facilitates surgery through its ease of performance and re-shortening capability (21). Although transverse osteotomy's stability has been questioned, the biomechanical study has revealed that the kind of osteotomy has no effect on the stability of the osteotomy site (22).

We analyzed the reasons for loosening after THA combined with subtrochanteric osteotomy and believe that the main reason is that the position of the subtrochanteric osteotomy is too low. Under the lesser trochanter, additional osteotomies are performed. The femoral isthmus and S-ROM prosthesis are not firmly fixed. There is no bone ingrowth in the distal femur, resulting in the inability to resist rotation under the S-ROM sleeve, which causes small movement between the prosthesis and the femur. Eventually, osteolysis, resorption, and loosening of the prosthesis occur at the osteotomy site (23). This is the primary cause of postoperative loosening, with secondary reasons including: (1) Due to osteotomy, bone union is absent or inadequate. (2) The excessive local dissection of the periosteum is caused by steel wire strapping. (3)Walk with a burden too early. (4) During osteotomy, the bone tissue's blood supply is compromised, resulting in nonunion of the osteotomy position.

Distal fixation was performed with tapered compression, with the femur in contact with the implant at least 20–50 mm. At least some of the femoral isthmus has to be spared (24), So prosthesis loosening following S-ROM arthroplasty is rare, and the main loosening site is located between the distal femur and the end of the S-ROM prosthesis. The submitted examples illustrate the surgical and medical difficulties associated with loosening after S-ROM arthroplasty. The repercussions are extremely severe. According to our treatment plan, the patient's hip joint recovered well after surgery, and the following is a summary of our treatment plan for prosthesis loosening: The prosthetic femoral implant was removed. If it was challenging to remove the proximal femur, and the greater trochanter osteotomy was performed and the S-ROM prosthesis was maintained. If the fracture is treated and the proximal femur is fixed, the surgeon needs to perform plate fixation and bone grafting. Through the report of three cases, we can better understand the main causes of postoperative loosening of S-ROM prosthesis and the treatment plan. After revision surgery for loosening of the S-ROM prosthesis, postoperative imaging, Harris score, and the patient's physical indicators indicated that the patients had recovered well.

## Conclusion

Prosthesis loosening after THA with Crowe IV DDH using an S-ROM prosthesis is a complex procedure, and we believe that the position of osteotomy under the lesser trochanter should not be too lower in the initial replacement. Generally, 1 cm–1.5 cm under the sleeve is better. A suitable length femoral stem must be selected, and the distal femur must be firmly fixed. If necessary, the distal femur should be connected with a steel wire to prevent distal femur fracture during hitting. Do not permit the patient to engage in early postoperative activity to prevent bone nonunion and loosening. If imaging examination confirms distal loosening of the prosthesis after THA and the proximal femur is fixed during the operation, then the femoral prosthesis fracture or nonunion is treated, and the plate fixation and bone grafting are performed. The patient recovered well after the revision.

## Data Availability

The raw data supporting the conclusions of this article will be made available by the authors, without undue reservation.
